# Long-Term Effect of Income Level on Mortality after Stroke: A Nationwide Cohort Study in South Korea

**DOI:** 10.3390/ijerph17228348

**Published:** 2020-11-11

**Authors:** Seungmin Jeong, Sung-il Cho, So Yeon Kong

**Affiliations:** 1 Department of Preventive Medicine, Kangwon National University Hospital, Chuncheon-si, Gangwon-do 24289, Korea; jdomini2@snu.ac.kr; 2 Department of Public Health Science, Graduate School of Public Health, and Institute of Health and Environment, Seoul National University, Seoul 08826, Korea; 3 Strategic Research, Laerdal Medical, 4002 Stavanger, Norway; Joyce.Kong@laerdal.com

**Keywords:** stroke, mortality, socioeconomic factors, healthcare disparities

## Abstract

We investigated whether income level has long-term effects on mortality rate in stroke patients and whether this varies with time after the first stroke event, using the National Health Insurance Service National Sample Cohort data from 2002 to 2015 in South Korea. The study population was new-onset stroke patients ≥18 years of age. Patients were categorized into Category (1) insured employees and Category (2) insured self-employed/Medical Aid beneficiaries. Each category was divided into three and four income level groups, retrospectively. The study population comprised of 11,668 patients. Among the Category 1 patients (*n* = 7720), the low-income group’s post-stroke mortality was 1.15-fold higher than the high-income group. Among the Category 2 patients (*n* = 3948), the lower income groups had higher post-stroke mortality than the high-income group (middle-income, aOR (adjusted odds ratio) 1.29; low-income, aOR 1.70; Medical Aid beneficiaries, aOR 2.19). In this category, the lower income groups’ post-stroke mortality risks compared to the high-income group were highest at 13–36 months after the first stroke event(middle-income, aOR 1.52; low-income, aOR 2.31; Medical Aid beneficiaries, aOR 2.53). Medical Aid beneficiaries had a significantly higher post-stroke mortality risk than the high-income group at all time points.

## 1. Introduction

Stroke is the second leading cause of death and a major cause of disability worldwide, which is the third-largest factor influencing disability-adjusted life years (DALYs) [1]. Despite significant advances in the treatment and prevention of stroke, there still remain striking disparities. It has long been recognized that significant socioeconomic disparities exist in stroke risk [2,3,4,5]. Many stroke patients suffer from reoccurrence, sequelae, and complications after the stroke [6,7]. Patients with a chronic condition like stroke often require long-term treatment and management for optimal outcomes and recovery, which may be prolonged with financial consequences. Therefore, stroke patients’ socioeconomic status (SES) or income level may be an important risk factor for long-term outcomes after stroke. Previous studies have reported SES differences in quality of stroke care and clinical outcomes after stroke [8,9,10]. In a Danish nationwide population-based study, stroke patients with low income and disability pension received substantially low quality of stroke care compared to high-income patients and employed patients [8]. In the study, higher risk of 30-day and 1-year case mortality was observed in unemployed patients compared to employed patients. The SES was also related to patients’ functional outcome, such as the modified Rankin scale, when the researchers evaluated two years after the stroke event [10]. Another population-based cohort study found a persistent survival advantage for stroke patients in the highest income level compared to those in the lowest income level [9]. While there may be a vulnerable phase during post-stroke period that stroke patients are more susceptible to adverse events, including mortality, the many of existing literature investigating post-stroke outcomes only focus on short-term outcomes of less than a year after the stroke [8,9,11], and no studies have investigated during which post-stroke period stroke patients are most vulnerable to mortality based on different income-levels of patients.

In this study, we used the National Health Insurance Service National Sample Cohort (NHIS-NSC) data from 2002 to 2015 to examine whether income level has long-term effects on mortality rate in stroke patients and whether this varies with time after the first stroke event. We also examined whether the medical benefits increased patients’ survival rate in the most impoverished economic strata receiving Medical Aid.

## 2. Materials and Methods

### 2.1. Study Setting

In South Korea, all citizens have access to universal health care and are required by law to enroll in the Healthcare Security System (HSS), which comprises National Health Insurance (NHI) and Medical Aid. Medical Aid benefits, similar to Medicaid in the USA, are provided to people in the lowest income category in South Korea. Generally, Medical Aid beneficiaries have lower incomes than the low-income group of the NHI [12]. Except for those supported by Medical Aid, all beneficiaries of the NHI are required to pay health insurance premiums. The NHI covers 97% of the Korean population, while Medical Aid beneficiaries accounted for the remaining 3% [12,13].

NHI is designed to protect insured employees and self-employed individuals. The insured employee category includes the employed individual’s dependents and immediate family, including siblings and parents from both sides of the family. The insured self-employed category includes those excluded from the insured employee category [12]. They pay monthly premiums to the HSS and are covered by NHI for defined medical treatments. However, for the treatments that are not defined in the NHI-approved items, the patient has to pay 100% for the treatment received directly to the hospital. To determine the premium, the government evaluates each household’s salary and assets rather than using the self-report.

Medical Aid is independently operated by the Korean government from the NHI. Medical Aid is a social safety net to secure the minimum livelihood of low-income households [14]. To qualify for Medical Aid, beneficiaries must be from a low-income household where the head of the household is unable to work. In such cases, medical treatment at a hospital is supported by the Korean government at little or no cost. Government-supported treatment is limited to the NHI-approved items. When the treatment is not included in the NHI-approved items, Medical Aid beneficiaries also need to pay 100% of the fee to the hospital directly [13].

Membership is determined by income level and the head of the household’s ability to work [12].

### 2.2. Data Source

In this study, data from the NHIS-NSC were used. This was a randomly selected cohort comprising 2.2% of the eligible South Korean population in 2006 and includes personal and demographic information, medical treatments from 2002 to 2015, and other pertinent information such as cause and time of death [15]. However, for the Medical Aid population, the medical treatment information from 2002 to 2005 was not included in this data. NHIS-NSC dataset provides the information where each individual is located in the decile of the insurance premium level. The premium calculating system varies between employees and self-employed. A recent study reported that the mean annual income of the employed category had $15,000 more annual income than the mean annual income of the self-employed category [16]. Thus, we categorized our study cohort into the employed group and the self-employed group. Furthermore, we combined the insured self-employed and Medical Aid beneficiaries into one category because most Medical Aid beneficiaries are unemployed [13].

### 2.3. Study Population

The inclusion criteria for this study were patients ≥18 years of age who visited emergency departments (EDs) for a first-ever stroke event in the NHIS-NSC data. Stroke was defined as the International Statistical Classification of Diseases and Related Health Problems, 10th Revision codes I60, I61, I62, and I63 [17].

Since the medical treatment information of the Medical Aid population from 2002 to 2005 were not available, the inclusion year criteria for each category were different. For the insured employee category, the stroke patients who visited EDs between 2004 and 2014 were included. For the insured self-employed/Medical Aid beneficiary category, stroke patients who visited EDs from 2008 to 2014 were included. Patients in both categories were followed until December 31, 2015. Washout periods of 2002–2003 for the employed category and 2006–2007 for the self-employed/Medical Aid category were used to exclude patients who may have been visiting EDs for the recurrence of stroke during the study period.

### 2.4. Variables

The main exposure variable was the income level. As a proxy for the income level, we used the insurance premium level. Both employed and self-employed groups were divided into three income groups. The high-income group included the top ~30% of patients by the income level, the low-income group included the bottom ~30% of patients by the income level, and the middle-income group included the remainder. The index year of the income-level was the year of the first stroke event. Since we combined the insured self-employed and Medical Aid beneficiaries into one category, this category had four groups for income level: High-income, middle-income, low-income, and the Medical Aid beneficiaries. The primary endpoint was post-stroke mortality. For those with post-stroke mortality, we calculated the time to death from date of stroke occurrence and the month of death. The NHIS-NSC uses the National Death Registration for patients’ death information, therefore we had no censored patients in regard to missing death information. Nine patients with no record of death (those who survived throughout the study period) were censored at the end of the study period (31 December 2015).

We categorized time of death into four periods:

- <3 months from the first stroke event
- 3–12 months after the first stroke event
- 13–36 months after the first stroke event
- >36 months after the first stroke event.

The confounding factors were as follows: age at the first stroke event; gender; physical disability status (none, mild, or severe) in the year of the first stroke event; location of the ED visited (metropolitan, urban, or rural); year of the first stroke event; Charlson Comorbidity Index (CCI) (≤2, 3–5, or >5); type of stroke (hemorrhagic, ischemic, or mixed); and the interaction between age and year of the first stroke event. The CCI was estimated using ICD-10 codes claimed during the two years before the date of the stroke occurrence [18].

### 2.5. Statistical Analysis

Baseline characteristics were described with frequency and percentage. Survival curves were made for employed and self-employed/Medical Aid groups, respectively, by different income levels, using the Kaplan-Meier method, adjusted for the confounding factors described above. The insured employee category was also adjusted on whether the patient is an employee or a family member of an employee. For the Cox regression analysis, we evaluated the proportional hazard assumption using a test based on the scaled Schoenfeld residuals. This assumption was satisfied for the insured employees (*p* = 0.89), and the multivariable Cox regression model was applied. However, the proportional hazard assumption was not satisfied with the insured self-employed/Medical Aid beneficiaries (*p* = 0.05). When the proportional assumption is not fulfilled, a Cox regression analysis yields inaccurate results, which can be overcome by applying other statistical methods [19,20,21]. Therefore, we used the method of Lindholt, which involves separate multinomial logistic regression analyses for the different periods for time of death since first-ever stroke [19]. The analyses were performed using SAS Enterprise Guide ver. 7.1 (SAS Institute Inc., Cary, NC, USA.) and R software (The R Foundation, Vienna, Austria).

### 2.6. Ethics Statement

This study was approved by the Institutional Review Board of Seoul National University (IRB No. E1808/003-002).

## 3. Results

During the study period, a total of 11,668 patients visited EDs with their first-ever stroke. Of these, 7720 patients were under the insured employee category, and were followed up for up to 11 years with a total of 38,148.6 person-years of follow-up. For the self-employed/Medical Aid beneficiary category a total of 3948 patients were enrolled and were followed up for up to 7 years with a total of 13,527 person-years of follow-up.

Table 1 shows the demographics of the study population by the employment status category. In the insured employee category, the proportion of male patients increased from high-income (50.0%) to low-income group (56.0%), while the proportion of male patients decreased from high-income (54.9%) to the low-income (43.0%) and Medical Aid beneficiaries (42.3%) groups in the self-employed/Medical Aid beneficiaries category. Patients in the low-income groups in the self-employed/Medical Aid category were in general, older (40.9% of low-income and 44.7% of Medical Aid beneficiaries patients in the age group of 65–80 years) and had higher Charlson Comorbidity Index (CCI) (48.6% for low-income and 62.9% in Medical Aid beneficiaries with CCI ≥6) compared with those in the low-income group of the employed category (48.9% were in the age group of 45–64 years and 36.3% with CCI ≥6). In the employed category, the post-stroke mortality rate was 36.7% for those in the high-income group and 28.0% for those in the low-income group. In the self-employed/Medical Aid beneficiary category, the post-stroke mortality rate was 25.0% for the high-income patients, 41.4% for low-income, and 47.8% for those in the Medical Aid beneficiary group.

**Figure 1:**
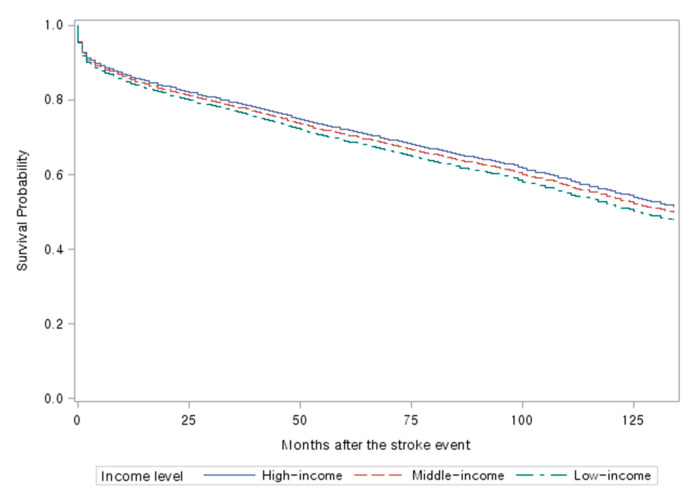
Multivariable adjusted survival Curves for Insured Employees by Income Level (*n* = 7720; 38,148.6 person-years). Adjusted for age, gender, physical disability status, location of the visited emergency department, year of the first stroke event, Charlson Comorbidity Index, type of stroke, the interaction between age and year of the first stroke event, and whether the patient is an employee or a family member of an employee.

**Figure 2:**
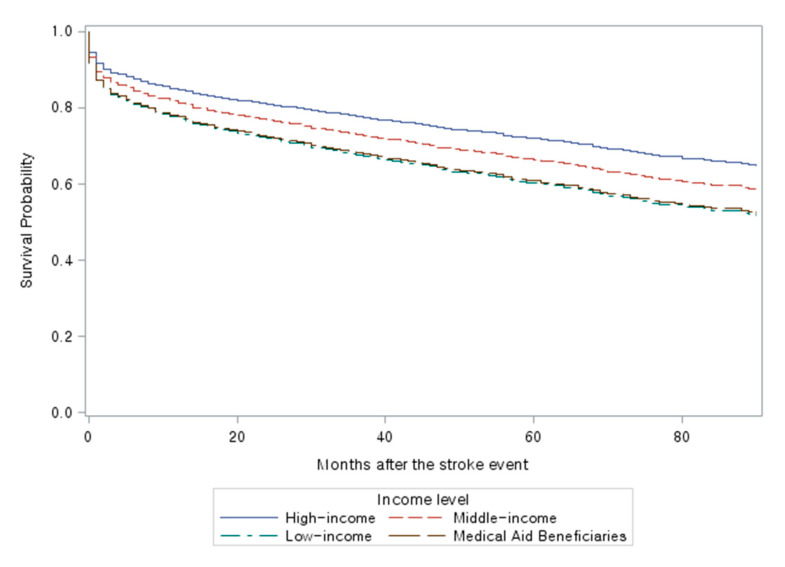
Survival Curves (Adjusted) for Insured Self-employed/Medical Aid Beneficiaries by Income Level (*n* = 3948; 13,527 person-years). Adjusted for age, gender, physical disability status, year of the first stroke event, Charlson Comorbidity Index, type of stroke, and the interaction between age and year of the first stroke event.

Figure 1 and Figure 2 are multivariable adjusted Kaplan–Meier survival curves of insured employees (Figure 1) and insured self-employees/Medical Aid beneficiaries (Figure 2) by income levels. In both categories, the lower income group showed a lower survival. However, the survival curves in the self-employed/Medical Aid beneficiaries category showed broader gaps among the high-, the middle-, and the low-income groups than those in the employed category. In the self-employed/Medical Aid beneficiary category, survival rates for the low-income group and the Medical Aid beneficiaries group were almost identical.

Table 2 describes the multivariable Cox regression to examine association between income level and post-stroke mortality among the insured employee patients. In the insured employee category, the low-income group had a mortality rate 1.15-fold (95% CI 1.04–1.28) that of the high-income group (*p* = 0.01), and the middle-income group had a mortality rate 1.06-fold (95% CI 0.97–1.17) that of the high-income group (*p* = 0.18).

The results of a multinomial regression analysis of the insured self-employed/Medical Aid beneficiary category are shown in Table 3. Using the high-income group as a reference, the middle-income group had a 1.38-fold higher total mortality risk (aOR 1.12–1.70), and the low-income group and Medical Aid beneficiaries group had 1.88-fold (aOR 1.45–2.44) and 2.06-fold (aOR 1.62–2.62) higher total mortality risks, respectively. In all different time periods for post-stroke mortality, we observed a statistically significant increase risk of post-stroke mortality in Medical Aid beneficiary groups compared with those in the high-income group. The highest post-mortality risk was observed in the Medical Aid beneficiaries group at 13–36 months after initial stroke with a 2.27-fold (aOR 1.66-3.85) greater risk for dying in Medical Aid beneficiaries group compared with those in the high-income group. For the low-income groups, the mortality risk was significantly higher than the high-income group up to 36 months following the first stroke. After 36 months, the increased risk of mortality was not statistically significant.

## 4. Discussion

In this study, we found that low-income stroke patients had a significantly higher risk of mortality than high-income stroke patients in the insured employee category. In the insured self-employed/Medical Aid beneficiary category, while a lower income was linked to a higher risk of mortality, this association was varied with both income level and time since the first stroke event. Moreover, the magnitude of difference in the risk of mortality between lower-income and high-income groups in the insured self-employed/Medical Aid beneficiary category was significantly greater than that in the insured employee category. Compared with the high-income reference group, the mortality risk of low income was highest at 13–26 months after the first stroke event, being 1.52-, 2.31-, and 2.53-fold higher in the middle-income, low-income, and Medical Aid beneficiaries groups, respectively.

Previous studies have yielded mixed findings on the effect of income level on the post-stroke mortality rate. Our results are in line with prior reports that socioeconomic status impacts post-stroke mortality [2,3,4,5]. Low-income patients do not receive the same level of care or have access to up-to-date diagnostic tools and treatments as higher-income patients [8,22,23]. The low-income is also related to health behaviors such as smoking, obesity, and physical inactivity, which have an adverse effect on stroke [24,25]. Several previous studies conducted in countries with well-established universal health care systems such as Canada, Denmark, and the United Kingdom, have found no significant relationship between income level and the mortality rate of stroke [5,8,26]. Even though all South Koreans have access to the universal health care system, the proportion of out-of-pocket payments is higher than the other OECD countries with universal health care systems [27]. Therefore, the significant relationship between income level and the mortality rate in stroke patients we observed in our study may be due to the lack of healthcare many low-income and Medical Aid patients received because they could not afford the costs of care [28].

Because we had a longer follow-up period than in most previous studies, our study had the strength to find more definitive evidence that income-related mortality varies over time. Lindmark et al. compared the mortality rate at 8–28 and 29–365 days after the first stroke event [11]. In each of these periods, the risk of mortality in the high-income group was 0.714- and 0.766-fold compared with that of the low-income group. Langagergaard et al. also found that income level was not significantly related to the mortality rate at 30 days and 1 year after the first stroke [8]. However, they did not evaluate the mortality rate beyond 1 year. The study by Rouling in South London involved follow-up for 17 years after the first stroke event and found no significant difference in mortality rate according to income level [26]. In contrast, our results suggested a relationship between mortality rate and income level. We consider this to be in part the result of differences in the maturity of the respective universal healthcare systems. Elfassy assessed mortality for an average of 5 years in the United States and found that, over time, low-income stroke patients had a proportionately higher mortality rate than high-income patients [29]. In contrast, our results for the insured self-employed/Medical Aid beneficiaries did not meet the proportional assumption criteria and indicated variations in mortality rate over time by income level. This could be due to the larger population (3948 subjects) in our study than the study by Elfassy (1329 subjects), which increases the sensitivity to minor variations in the data.

In our study, the proportional assumption for the insured employees was satisfied despite the large number of participants (7720 subjects). In this category, the hazard ratio for mortality in the low-income group, compared with the high-income group, was less than that of the insured self-employed. This is likely because the income distribution is more uniform among the insured employees than among the insured self-employed, and the average income of the insured employees is higher than that of the insured self-employed [16].

Among the insured self-employed/Medical Aid beneficiaries, we observed the highest mortality rate in the 13–36 months following the first stroke event. This is a critical period when the risk of complications of stroke (such as infection and recurrent stroke episodes) is high [7]. In recurrent episodes, the lower-income patients and their families often have financial difficulty to pay for additional treatment after paying for the first hospitalization and treatment. Complications should be managed continuously; however, financial hardship may result in the abandonment of treatment by Medical Aid beneficiaries with a low income.

Although Medical Aid beneficiaries received the highest proportion of medical benefits from the government when admitted to hospital, they had the highest mortality risk for all time points. This implies that receiving Medical Aid alone does not lead to a reduction in mortality. There are three reasons for this. First, the problems of care cannot be solved by support for hospital expenses alone because they worsen over time, especially when complications arise. Second, Medical Aid beneficiaries are only supported for NHIS-approved items and, as such, are required to pay 100% of the cost of non-approved treatments. Some treatments, particularly new treatments, are not yet approved by the NHIS for the treatment of stroke. Therefore, Medical Aid beneficiaries requiring these treatments are less likely to be qualified for benefits. Third, patients of lower SES tend to have a longer onset-to-door time; therefore, their condition may be worsened by the time they receive treatment [30,31,32].

This study has a number of strengths. First, we used data from the NHIS-NSC, which is a representative of the national population with the same health insurance system. Second, only 13 of the 11,681 participants moved away or were lost to follow-up, which was a very insignificant number. Third, while most previous studies used indirect indicators, such as a regional zip code, in measuring income level [4,5,33], we used the actual insurance premium decile, which more accurately reflects individual patient’s income level.

With these strengths, beyond the previous studies, we could find out that the income inequity on post-stroke mortality was the highest in the 13–26 months after the first stroke event. This study also provides evidence that the benefit of Medical Aid alone is not sufficient for stroke patients’ care. Therefore, our findings can support the need for other kinds of assistance, such as a caregiver dispatch program and cash aid program.

This study also has limitations. First, information on several factors that may contribute to poor outcomes, including patients’ accompanying diseases and education level were not available in this study. Also, there was a possibility of unmeasured confounding effects, which could not be easily incorporated into the study. Second, the NHIS-NSC did not include information on clinical conditions such as blood pressure, consciousness, or National Institute of Health Stroke Scale rating at the time of the ED visit; thus, we could not adjust for the severity of the first stroke event. Third, while income level can change over time, we analyzed the income level at the time of stroke. Fourth, due to the format of the data, only the month of death could be confirmed. We were unable to confirm the exact day or precise time of death. Thus, it was not possible to assess the mortality rate according to income level in the hyperacute period within 1 month of the first stroke event. Fifth, this study was conducted in South Korea; therefore, other cultural/geographical conditions could have different results.

Further studies considering the change of the income-level or using the data with the hyperacute period information could usefully explore how the income-level works on post-stroke mortality. Also, studies conducted in other countries could provide evidence for the external validity of our results.

## 5. Conclusions

The mortality risk of low-income stroke patients was higher than that of the high-income stroke patients. For the self-employed/Medical Aid beneficiaries, the mortality risk varied over time, peaking at 13–36 months after the first stroke event. There was no evidence of increased survival benefits in the group receiving Medical Aid. This study demonstrates the need for support of low-income stroke patients, particularly in the 13–36 months after the first stroke event. The study results also suggest that Medical Aid beneficiaries require active supports and aids.

## Figures and Tables

**Table 1:** Demographics of the Study Population.

Demographic Characteristics	Employees				Self-Employed and Medical Aid Beneficiaries				
	Total	High-Income	Middle-Income	Low-Income	Total	High-Income	Middle-Income	Low-Income	Medical Aid Beneficiaries
Total *n* (%)	7720 (100)	2534 (100)	2964 (100)	2222 (100)	3948 (100)	799 (100)	1826 (100)	553 (100)	770 (100)
Gender *n* (%)									
Male	4109 (53.2)	1268 (50.0)	1596 (53.9)	1245 (56.0)	2074 (52.5)	439 (54.9)	1071 (58.7)	238 (43.0)	326 (42.3)
Female	3611 (46.8)	1266 (50.0)	1368 (46.1)	977 (44.0)	1874 (47.5)	360 (45.1)	755 (41.4)	315 (57.0)	444 (57.7)
Age *n* (%)									
18–44 years	694 (9.0)	167 (6.6)	304 (10.3)	223 (10.0)	362 (9.2)	70 (8.8)	195 (10.7)	51 (9.2)	46 (6.0)
45–64 years	2715 (35.2)	573 (22.6)	1055 (35.6)	1087 (48.9)	1563 (39.6)	352 (44.1)	815 (44.6)	165 (29.8)	231 (30.0)
65–80 years	3303 (42.8)	1305 (51.5)	1290 (43.5)	708 (31.9)	1517 (38.4)	295 (36.9)	652 (35.7)	226 (40.9)	344 (44.7)
>80 years	1008 (13.0)	489 (19.3)	315 (10.6)	204 (9.2)	506 (12.8)	82 (10.3)	164 (9.0)	111 (20.1)	149 (19.4)
Physical Disability Status *n* (%)									
Severe	164 (2.1)	63 (2.5)	65 (2.2)	36 (1.6)	166 (4.2)	14 (1.8)	40 (2.2)	24 (4.3)	88 (11.4)
Mild	701 (9.1)	225 (8.9)	286 (9.7)	190 (8.6)	453 (11.5)	63 (7.9)	164 (9)	65 (11.8)	161 (20.9)
None	6855 (88.8)	2246 (88.6)	2613 (88.1)	1996 (89.8)	3329 (84.3)	722 (90.4)	1622 (88.8)	464 (83.9)	521 (67.7)
Living Location *n* (%)									
Rural	1030 (13.3)	373 (14.7)	426 (14.4)	231 (10.4)	621 (15.7)	79 (9.9)	302 (16.5)	109 (19.7)	131 (17.0)
Urban	3390 (43.9)	1080 (42.6)	1296 (43.7)	1014 (45.6)	1695 (42.9)	336 (42.1)	786 (43)	233 (42.1)	340 (44.2)
Metropolitan	3300 (42.7)	1081 (42.7)	1242 (41.9)	977 (44.0)	1632 (41.3)	384 (48.1)	738 (40.4)	211 (38.2)	299 (38.8)
Visited ED *n* (%)									
Rural	226 (2.9)	85 (3.3)	98 (3.3)	43 (1.9)	155 (3.9)	16 (2.0)	68 (3.7)	33 (6.0)	38 (4.9)
Urban	3462 (44.9)	1117 (44.1)	1341 (45.2)	1004 (45.2)	1800 (45.6)	315 (39.4)	856 (46.9)	260 (47.0)	369 (47.9)
Metropolitan	4032 (52.2)	1332 (52.6)	1525 (51.5)	1175 (52.9)	1993 (50.5)	468 (58.6)	902 (49.4)	260 (47.0)	363 (47.1)
Incidence Year of Initial Stroke Event *n* (%)									
2004–2006	1856 (24.0)	601 (23.7)	727 (24.5)	528 (23.8)					
2007–2010	3023 (39.2)	995 (39.3)	1192 (40.2)	836 (37.6)	1890 (47.9)	376 (47.1)	828 (45.3)	261 (47.2)	425 (55.2)
2011–2014	2841 (36.8)	938 (37.0)	1045 (35.3)	858 (38.6)	2058 (52.1)	423 (52.9)	998 (54.7)	292 (52.8)	345 (44.8)
CCI *n* (%)									
0–2	1375 (17.8)	328 (12.9)	539 (18.2)	508 (22.9)	698 (17.7)	154 (19.3)	399 (21.9)	85 (15.4)	60 (7.8)
3–5	2921 (37.8)	839 (33.1)	1176 (39.7)	906 (40.8)	1427 (36.1)	308 (38.5)	694 (38.0)	199 (36.0)	226 (29.4)
≥6	3424 (44.4)	1367 (54.0)	1249 (42.1)	808 (36.3)	1823 (46.2)	337 (42.2)	733 (40.1)	269 (48.6)	484 (62.9)
Type of the Stroke *n* (%)									
Hemorrhagic	2132 (27.6)	629 (24.8)	818 (27.6)	685 (30.8)	1194 (30.2)	238 (29.8)	613 (33.6)	160 (28.9)	183 (23.8)
Ischemic	5331 (69.1)	1827 (72.1)	2048 (69.1)	1456 (65.5)	2621 (66.4)	541 (67.7)	1145 (62.7)	369 (66.7)	566 (73.5)
Mixed	257 (3.3)	78 (3.1)	98 (3.3)	81 (3.7)	133 (3.4)	20 (2.5)	68 (3.7)	24 (4.3)	21 (2.7)
Visited Hospital Level *n* (%)									
Primary	430 (5.6)	150 (5.9)	161 (5.4)	119 (5.3)	253 (6.4)	38 (4.8)	108 (5.9)	37 (6.7)	70 (9.1)
Secondary	4035 (52.3)	1288 (50.8)	1530 (51.6)	1217 (54.8)	2240 (56.7)	382 (47.8)	1033 (56.6)	345 (62.4)	480 (62.3)
Tertiary	3255 (42.1)	1096 (43.3)	1273 (43.0)	886 (39.9)	1455 (36.9)	379 (47.4)	685 (37.5)	171 (30.9)	220 (28.6)
Clinical Outcome *n* (%)									
Survived	5225 (67.7)	1605 (63.3)	2021 (68.2)	1599 (72.0)	2611 (66.1)	599 (75.0)	1286 (70.4)	324 (58.6)	402 (52.2)
Died	2495 (32.3)	929 (36.7)	943 (31.8)	623 (28.0)	1337 (33.9)	200 (25.0)	540 (29.6)	229 (41.4)	368 (47.8)

ED—Emergency Department; CCI—Charlson Comorbidity Index.

**Table 2:** Results of Cox Regression Analysis for Insured Employees Patients.

Groups	Patients (*n*)	Death (*n*)	Mortality Rate (%)	aHR (95% CI)
High income	2534	929	36.7	Reference
Middle income	2964	943	31.8	1.06 (0.97–1.17)
Low income	2222	623	28.0	1.15 (1.04–1.28)

aHR—adjusted hazard ratio; CI—confidence interval.

**Table 3:** Results of Multinomial Logistic Regression for Self-employed Insured/Medical Aid Patients.

Groups	Self-Employed Insured/Medical Aid Patients	
	*n* (Mortality Rate%)	aOR (95% CI)
Total death	1337 (33.9%)	
High income	200 (25.0%)	Reference
Middle income	540 (29.6%)	1.38 (1.12–1.70)
Low income	229 (41.4%)	1.88 (1.45–2.44)
Medical Aid beneficiaries	368 (47.8%)	2.06 (1.62–2.62)
Within 3 months of initial stroke	578 (14.6%)	
High income	89 (11.1%)	Reference
Middle income	255 (14%)	1.37 (1.04–1.81)
Low income	95 (17.2%)	1.70 (1.20–2.39)
Medical Aid beneficiaries	139 (18.1%)	1.74 (1.26–2.41)
3–12 months after initial stroke	217 (5.5%)	
High income	32 (4.0%)	Reference
Middle income	79 (4.3%)	1.27 (0.83–1.97)
Low income	42 (7.6%)	2.15 (1.31–3.55)
Medical Aid beneficiaries	64 (8.3%)	2.27 (1.43–3.62)
13–36 months after initial stroke	293 (7.4%)	
High income	38 (4.8%)	Reference
Middle income	110 (6.0%)	1.52 (1.03–2.26)
Low income	55 (9.9%)	2.31 (1.47–3.64)
Medical Aid beneficiaries	90 (11.7%)	2.53 (1.66–3.85)
Over 36 months past initial stroke	249 (6.3%)	
High income	41 (5.1%)	Reference
Middle income	96 (5.3%)	1.27 (0.85–1.90)
Low income	37 (6.7%)	1.55 (0.95–2.55)
Medical Aid beneficiaries	75 (9.7%)	2.04 (1.33–3.15)

aOR—adjusted odds ratio; CI—confidence interval.
